# Developing a Consistent, Reproducible Botulinum Toxin Type A Dosing Method for Upper Limb Tremor by Kinematic Analysis

**DOI:** 10.3390/toxins13040264

**Published:** 2021-04-08

**Authors:** Olivia Samotus, Jack Lee, Mandar Jog

**Affiliations:** 1 Department of Clinical Neurological Sciences, London Health Sciences Centre—Lawson Health Research Institute, 339 Windermere Road, A10-026, London, ON N6A 5A5, Canada; mandar.jog@lhsc.on.ca; 2 Schulich School of Medicine and Dentistry, University of Western, 1151 Richmond Street, London, ON N6A 3K7, Canada; 3 MDDT Inc., London, ON N6G 0J3, Canada; jack.lee@lhsc.on.ca

**Keywords:** botulinum toxin, upper limb tremor, dosing algorithm, kinematics, computer-assisted dosing, clinical-decision support, treatment planning, injection pattern

## Abstract

Botulinum toxin type A (BoNT-A) injection patterns customized to each patient’s unique tremor characteristics produce better efficacy and lower adverse effects compared to the fixed-muscle-fixed-dose approach for Essential Tremor (ET) and Parkinson’s disease (PD) tremor therapy. This article outlined how a kinematic-based dosing method to standardize and customize BoNT-A injections for tremors was developed. Seven ET and eight PD participants with significant tremor reduction and minimal perceived weakness using optimized BoNT-A injections determined by clinical and kinematic guidance were retrospectively selected to develop the kinematic-based dosing method. BoNT-A dosages allocated per joint were paired to baseline tremor amplitudes per joint. The final kinematic-based dosing method was prospectively utilized to validate BoNT-A injection pattern selection without clinical/visual assessments in 31 ET and 47 PD participants with debilitating arm tremors (totaling 122 unique tremor patterns). Whole-arm kinematic tremor analysis was performed at baseline and 6-weeks post-injection. Correlation and linear regression analyses between baseline tremor amplitudes and the change in tremor amplitude 6-weeks post-injection, with BoNT-A dosages per joint, were performed. Injection patterns determined using clinical assessment and interpretation of kinematics produced significant associations between baseline tremor amplitudes and optimized BoNT-A dosages in all joints. The change in elbow tremor was only significantly associated with the elbow total dose as the change in the wrist and shoulder tremor amplitudes were not significantly associated with the wrist and shoulder dosages from the selected 15 ET and PD participants. Using the kinematic-based dosing method, significant associations between baseline tremor amplitudes and the change (6-weeks post-first treatment) in tremor at each joint with BoNT-A dosages for all joints was observed in all 78 ET and PD participants. The kinematic-based dosing method provided consistency in dose selection and subsequent tremor reduction and can be used to standardize tremor assessments for whole-arm tremor treatment planning.

## 1. Introduction

Debilitating upper limb tremor resistant to oral pharmacological interventions [1,2,3] can be treated using local injections of botulinum toxin type A (BoNT-A) in Essential Tremor (ET) and Parkinson’s disease (PD) patients [4,5,6]. Past studies using fixed-dosing regimens have shown that significant tremor reduction may be coupled with no functional improvements and intolerable side effects such as muscle weakness before treatment optimization [7,8,9]. Needle-guided techniques (e.g., electromyography [EMG], electrical stimulation), ultrasound, or surface EMG and anatomy are used for more accurate targeting of muscles contributing to tremor motion. Using such techniques aid the injector to select muscles for injection, which has improved functional outcomes and minimized excessive arm weakness [10,11,12,13,14,15,16,17]. However, there is no available software for tremor analysis to customize BoNT-A dosing for the selection of muscle groups without substantial clinical judgment [18,19]. Thus, these limiting aspects may reduce feasibility for use in real-world clinics and consistency in replicating a standardized approach of BoNT-A injection pattern determination [6]. Newer comprehensive technology-based injection techniques, such as the Yale method (EMG needle-probing of forearm muscles) [10,11] and whole-arm kinematic tremor assessments by Jog et al. [12,13,14,15] can now guide clinical customization of BoNT-A dose and muscle selections based on each patient’s tremor characteristics [19]. Both BoNT-A dosing techniques have similarly demonstrated significant tremor reduction, improved motor function, and quality of life while significantly reducing the incidence of muscle weakness in tremulous ET and PD patients [20]. The Yale method that focuses only on forearm muscle groups does require greater expertise to perform needle EMG and interpret EMG signals and is time-consuming, costly, and painful. The whole-arm kinematic-based dosing method is a non-invasive motion-sensor assessment that can be performed by a medical assistant in approximately 15 to 20 min including sensor placement, and tremor data analysis to support the clinician’s final dosing calculations. Determination of injection patterns can be performed by any injector with experience of upper limb anatomy and does not require engineering proficiency as tremor characteristics (amplitude and directional contribution of tremor per joint) are analyzed and easily interpreted. A 30-min training course is required to learn how to use the sensors (Biometrics Ltd.) and tremor analysis software (TremorTek^®^ currently undergoing commercialization, MDDT Inc.). Furthermore, the kinematic-based approach to aid BoNT-A dosing has been validated in two pilot studies serially treating ET and PD patients [13,14] and replicated in a double-blinded, placebo-controlled, multi-centered clinical trial involving a single-injection in ET patients [15].

Whole-arm kinematic tremor analysis measures tremor severity at each arm joint and further distinguishes the contribution of tremor in each degree of freedom acting per arm joint, including tremor asymmetry such as wrist tremor bias. Kinematics can be used to monitor and optimize injections by measuring the change in tremor severity following treatments. Thus, this research article outlined how the kinematic-based dosing method was developed for its use in these two prospective pilot studies [13,14] and was replicated in the prospective multi-center clinical trial [15]. ET and PD patients with significant tremor relief and minimal muscle weakness treated using optimized (maintained after the fourth serial treatment) injection patterns determined by clinical and kinematic assessments were retrospectively used to establish dose selection based on baseline tremor severity [12]. By validating the kinematic-based dosing method in the second cohort of ET and PD patients [13,14], a relationship between the change in tremor severity and BoNT-A dosages allocated to muscle groups acting upon each arm joint was investigated.

## 2. Results

### 2.1. Dosing Method Development and Validation

**Figure 1:**
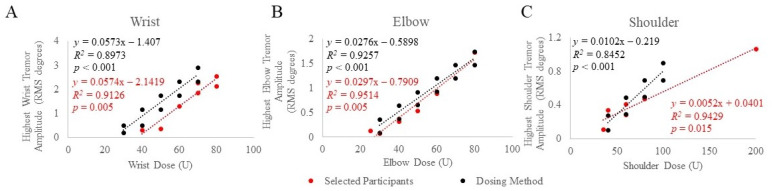
Mean baseline tremor amplitude was plotted against BoNT-A dosages allocated to the wrist (**A**), elbow (**B**), and shoulder (**C**) joints in selected participants (red) treated using optimized injection patterns determined by clinical and kinematic guidance and the final established dosing method (black). The scripted task that produced the highest tremor amplitude was plotted against joint dosages optimized from participants with significant tremor reduction and minimal perceived weakness. *p*-values < 0.05 indicate significant correlations using Spearman’s rho statistical test.

A range of baseline tremor amplitudes at each joint corresponding to BoNT-A joint dosages that produced significant tremor reduction with minimal perceived weakness in a retrospective selection of ET and PD participants were established using injection patterns determined by clinical and kinematic guidance [12]. Selected participants’ baseline tremor amplitudes, from the task that produced the highest amplitude, were plotted against joint dosages that were optimized (stayed the same from the fourth treatment and onwards), as illustrated in Figure 1A–C. Significant Spearman rho’s correlations between optimized BoNT-A dosages and baseline tremor amplitudes at the wrist (*r_s_*(7) = 0.909, *p* = 0.005) elbow (*r_s_*(6) = 0.943, *p* = 0.005) and shoulder (*r_s_*(6) = 0.899, *p* = 0.015) joints were demonstrated. Based on these results, a dosing method relating baseline tremor amplitudes and BoNT-A dose per joint was established. Using the best clinical judgment to further minimize the likelihood of wrist/hand muscle weakness, a 10 U decrease in wrist dosages was utilized in the final kinematic-based dosing method (Figure 1A).

**Figure 2:**
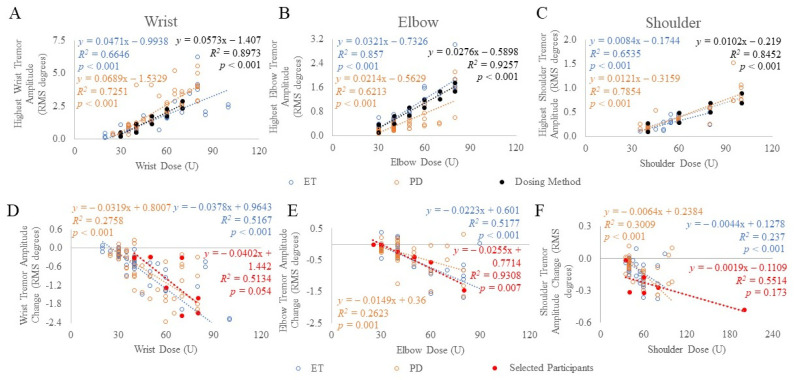
Mean tremor amplitude (**A**–**C**) and mean change in tremor amplitude (**D**–**F**) was plotted against BoNT-A dosages allocated to each arm joint in ET (blue) and PD (orange) participants treated using kinematic-based dosing method (black), and selected participants (red) treated using clinical and kinematic guided, optimized dosages. The scripted task that produced the highest tremor amplitude at each arm joint was plotted. *p*-values < 0.05 indicate significant correlations using Spearman’s rho statistical test.

Using the established kinematic-based dosing method, a second cohort totaling 78 ET and PD participants [13,14] were allocated dosages based on the task that produced the highest tremor amplitude per arm joint (Figure 2A–C). Thus, the minimum and maximum BoNT-A dosages allocated to the wrist, elbow, and shoulder joints were 30 to 70 U, 30 to 80 U, and 40 to 100 U, respectively, totaling a total dose ranging between 30 to 250 U per arm. These joint dosages were allocated to muscles, selected by the injector, depending on the percent contribution of tremor using Table 2. For example, if a wrist dose of 30 U was required based on the wrist tremor amplitude (“dosing method” as plotted in Figure 1 and Figure 2A–C), and flexion-extension (F/E) and radial-ulnar (R/U) tremor contributions were 50% and 15%, respectively, then based on Table 2 and rounding to the nearest 5 U, 5 U per muscle from the total 30 U wrist dose would be allocated to the FCR, FCU, ECR, and ECU muscle groups. The injector can also distribute the dose amongst these muscles based on clinical interpretation of wrist bias outputted from the tremor analysis. This leaves 35% of the tremor in the pronation-supination (P/S) direction resulting in 5 U allocated to each of the PT, PQ, and supinator muscles. Due to rounding, the total wrist dose is now 35 U, thus, to match the 30 U dose cap for the first injection, the ECR dose was reduced by 5 U. Results of the correlation analysis (Spearman-rho) demonstrated a significant correlation between BoNT-A dosages and tremor amplitudes at the wrist (ET: *r_s_*(62) = 0.901, *p* < 0.001 and PD: rs(60) = 0.885, *p* < 0.001), elbow (ET: *r_s_*(62) = 0.830, *p* < 0.001 and PD: *r_s_*(60) = 0.915, *p* < 0.001) and shoulder (ET: *r_s_*(62) = 0.698, *p* < 0.001 and PD: *r_s_*(60) = 0.835, *p* < 0.001) arm joints (Figure 2A–C).

### 2.2. Associations between Tremor Reduction and Total Joint Dose

In the selected participant cohort optimally treated using clinical and kinematic guidance, the change in tremor amplitude following the fourth serial treatment compared to baseline tremor amplitudes was significantly correlated (Spearman-rho) at the elbow (*r_s_*(6) = 0.893, *p* = 0.007), but not at the wrist (*r_s_*(7) = 0.746, *p* = 0.054) or shoulder joints (*r_s_*(6) = 0.638, *p* = 0.173) (Figure 2D–F). In the second ET and PD cohort treated using the kinematic-based dosing method, the change (6-weeks post-injection) in tremor amplitude following the first injection was significantly correlated to BoNT-A dosages allocated to the wrist (ET: *r_s_*(62) = −0.855, *p* < 0.001 and PD: *r_s_*(60) = −0.617, *p* < 0.001), elbow (ET: *r_s_*(62) = −0.657, *p* < 0.001 and PD: *r_s_*(60) = −0.444, *p* = 0.001) and shoulder (ET: *r_s_*(62) = −0.642, *p* < 0.001 and PD: *r_s_*(60) = −0.604, *p* < 0.001) joints (Figure 2D–F).

The linear regression model established a significant relationship between the change in tremor amplitude per joint and the BoNT-A dose allocated per joint in the selected participants treated by the clinical and kinematic-guided method. The model revealed an increase in BoNT-A dose by 10 U was associated with a reduction in elbow tremor amplitude by β = 0.026 (*p* = 0.002). There were no significant associations between the change in the wrist (*p* = 0.07) or shoulder amplitudes (*p* = 0.091) with the BoNT-A dose. For ET and PD participants treated using the kinematic-based method, the model revealed an increase in BoNT-A dose by 10 U was associated with a reduction in wrist tremor amplitude by β = 0.38 (*p* < 0.001) and β = 0.32 (*p* < 0.001) RMS degrees in ET and PD cohorts, respectively. The model revealed an increase in BoNT-A dose by 10 U was associated with a reduction in elbow tremor amplitude by β = 0.22 (*p* < 0.001) and β = 0.15 (*p* < 0.001) RMS degrees in ET and PD cohorts, respectively. The model revealed an increase in BoNT-A dose by 10 U was associated with a reduction in shoulder tremor amplitude by β = 0.04 (*p* < 0.001) and β = 0.06 (*p* < 0.001) RMS degrees in ET and PD cohorts, respectively.

## 3. Discussion

Variations in tremor amplitude and direction within multiple arm joints simultaneously make a visual assessment of whole-arm tremor challenging. Thus, many injectors choose to not treat upper limb tremors using BoNT-A injections as improper assessment leads to poor dose and muscle selections ultimately causing disabling weakness. Feasibility in the use of needle-based or sensor-based tremor assessment techniques to aid injectors in the clinic requires developed and tested software [15]. This unmet need can be addressed by a comprehensive, validated approach measuring tremor amplitude and direction and using such tremor analysis to base BoNT-A dose selection to muscle groups along the whole arm [13,14,15]. Thus, in this article, a linear relationship between dose selection and tremor severity per joint was established from participants with significant tremor reduction and minimal weakness using injection patterns optimized by integrating visual assessments and clinical interpretation of kinematics. Subsequently, the dosing method was established and validated in a second cohort of toxin-naïve ET and PD participants treated using kinematic-based injections without participants requiring any clinical assessment of their arm tremor (amplitude or direction). Using the kinematic-based dosing method, significant association in tremor reduction and BoNT-A dose was achieved along the whole arm. This demonstrated consistency in dosing and subsequent tremor reduction using the standardized kinematic-based dosing method for patients with mild to severe tremors. However, this was not observed in the initially selected participant cohort who were dosed using both clinical/visual assessment and kinematic guidance (clinical interpretation of the kinematic tremor analysis). Thus, when relying on the injector’s gestalt for visual assessment and data interpretation, increased variability and inconsistency in dose selection and tremor outcomes may occur.

This kinematic-based dosing method has been replicated in a recent double-blinded, placebo-controlled, multi-centered clinical trial involving a single-injection in ET patients [15]. This clinical trial demonstrated a significant reduction in tremor severity in the treatment group which utilized ~80% of injection patterns solely based on kinematic analysis. As previously published, tremor amplitude was significantly reduced after the first treatment and was maintained over three serial treatments (every 3 months) [13,14]. However, a limitation of using kinematics is the inability to precisely pinpoint individual tremulous muscle activity that contributes to the tremor motion within each plane of motion. For example, kinematic results show 35% of wrist tremor contribution in the pronation-supination (P/S) direction, the dosing method would recommend injecting the agonist and antagonist muscle groups, pronator quadratus, pronator teres, and the supinator, predominantly generating the rotational motion based on clinical judgment. The combination of using surface EMG and kinematics along the whole-arm could improve accuracy in targeting superficial tremulous muscles. However, the additional benefits in tremor reduction and tolerability, and the feasibility, cost, and time to conduct both surface EMG and kinematics have yet to be proven and warrants further investigation.

This kinematic-based dosing method can be utilized as a guide for a clinician/injector to use the muscles previously reported [13,14,15]. The dosing method can be modifiable as clinicians can choose to add/remove muscles based on their approach as to which muscles are contributing to joint motion [19]. For example, brachioradialis for elbow flexion can be injected in addition to the biceps. Similarly, clinicians can further modify dosage allocations based on wrist posturing/bias during anti-gravity tasks (arms outstretched with palms facing downwards (“Posture-1”) or facing inwards (“Posture-2”)) that can be extracted kinematically. For example, if a patient has a radial wrist tremor posturing, 5 U can be removed from the ulnar muscles and added to the radial wrist muscle groups. The sensors used for kinematic tremor assessments are commercially available (Biometrics Ltd.). The software (TremorTek^®^; MDDT Inc.) including data acquisition and analysis of tremors during different scripted tasks, and the dosing method can aid the injector to improve consistency and reproducibility in tremor treatment planning and outcomes. TremorTek^®^ is currently available to purchase for research purposes and is undergoing commercialization. The dosing method can be developed into a clinical decision support software to guide injectors in selecting optimal BoNT-A dosages relating to a patient’s tremor characteristics. Future clinical trials may investigate the feasibility, efficacy, and tolerability of using BoNT-A injection parameters based on dosing techniques that are determined without the aid or interpretation of an expert injector against other technology-based treatment methods that require the injector’s judgment.

This study demonstrated that there was a significant relationship between tremor reduction and the BoNT-A dosages administered when injection patterns were determined using the kinematic-based dosing method. The dosing method was developed based on the clinical interpretation of kinematic tremor analysis. Kinematics paired with the dosing method can standardize both the assessment of tremor and BoNT-A dose selection to muscle groups predominantly contributing to the tremor motion. Final injection patterns still require confirmation with the injector but the dosing method facilitates optimal dosages required to treat different tremor severities without producing excessive arm weakness [13,14,15]. Currently, already approved indications of BoNT-A for other movement disorders such as cervical dystonia (CD) suffer from variability in efficacy due to poor assessment and dosing [21,22]. With increasing accessibility to wearable sensors and smart technology, the dosing method developed has been successfully applied to the treatment of CD [23]. Our approach for utilizing kinematics to develop a dosing method is critical when initiating and optimizing BoNT-A therapy in conditions where multiple joints or complex movements are involved.

## 4. Materials and Methods

### 4.1. Study Participants

To establish the dosing method, study results including kinematic tremor analysis at each arm joint and the optimized (no change) dosing patterns from a selected cohort of ET and PD participants who completed the open-label, single-center, single injector, prospective 96-week study (REB#18445, clinicaltrials.gov (accessed on 6 April 2021) registry Identifier: NCT02427646) were retrospectively selected [12]. This study utilized dosing patterns for each arm joint that were determined using clinical and kinematic assessments (clinical interpretation of the kinematic tremor analysis in addition to visual assessments) [12]. The total dose per joint were calculated by adding the dosages for muscles acting upon each joint. Of the 32 participants who completed the 96-week [12], 7 ET and 8 PD participants were retrospectively selected as these participants had significant tremor relief and minimal perceived muscle weakness using optimized injection patterns starting at the fourth serial treatment cycle (week 48) and were maintained till the last (sixth) serial treatment. These 15 selected participants’ kinematic data and corresponding injection patterns were utilized to develop the dosing method. None of these participants were included in the second cohort of participants who were treated using the kinematic-based dosing method [13,14].

Whole-arm dosing patterns per participant were not selected as the tremor was not present in all joints for all participants. The optimal dose related to the reduction in tremor severity at each joint was used for the dosing method (wrist *n* = 7, elbow and shoulder *n* = 6 each) (Table 1). Participant demographics of the selected 7 ET and 8 PD participants are shown in Table 1. Kinematic data from baseline (week 0) and 6-weeks following the fourth treatment were plotted against optimized BoNT-A dosages per joint.

Validation of the kinematic-based dosing method was demonstrated from a convenience sampling of 31 ET and 47 PD participants from the prospective, open-label, single-center, single injector studies approved by the Western University Health Sciences Research Ethics Board (REBs #107433 and #104584) and was registered in the clinicaltrials.gov (accessed on 6 April 2021) registry (NCT02551848 and NCT02668497). None of the 31 ET [13] and 47 PD [14] participants used to validate the dosing method were included in the retrospectively selected 15 participants from the prospective 96-week study [12]. All 31 ET participants were treated bilaterally. In the PD cohort, 13 participants were treated bilaterally, and 34 participants were treated unilaterally. Thus, 62 data points (injection pattern per limb) in the ET cohort and 60 data points in the PD cohort, totaling 122 unique tremor injection patterns were utilized; participant demographics were previously reported [13,14]. Total joint BoNT-A dosages were calculated from injection patterns determined using the dosing method (linear relationship between total dose per joint and joint tremor amplitudes): (1) wrist: minimum = 30 U corresponded to >0.1 RMS degrees and maximum = 80 U corresponded to >2.32 RMS degrees, (2) elbow: minimum = 30 U corresponded to >0.1 RMS degrees and maximum = 80 U corresponded to >1.47 RMS degrees, (3) shoulder: minimum = 40 U corresponded to >0.1 RMS degrees and maximum = 100 U corresponded to >0.7 RMS degrees. For each participant, the task to produce the highest tremor severity at each joint was used for dose and muscle selection. Mean tremor amplitudes ≤ 0.1 RMS degrees were not utilized for dosing. Dosages allocated to muscle groups acting at each arm joint were based on tremor severity. Dosages were divided into muscle groups depending on the directional separation of tremors at each arm joint, as displayed in Table 2. Individual muscle dosages injected in wrist/forearm, elbow, and shoulder muscle groups ranged from 5 to 20 U, 15 to 40 U, and 10 to 50 U, respectively [13,14]. Up to 13 muscles were selected by the clinician to be treated and dosages were rounded to the nearest 5 U. Participants underwent kinematic assessments at week 0 and week 6, following the first treatment [13,14].

The ethics committee provided full board approval for all clinical trial protocols, and written consent was obtained from all participants recruited from the London Movement Disorders Centre in London, Ontario, Canada. For all participants, tremor was functionally debilitating and was their most bothersome symptom. All participants were treated with BoNT-A (incobotulinumtoxinA; Xeomin^®^, Merz Pharma) diluted in 0.9% saline without preservative and diluted to a concentration of 20 U per 0.1 mL. All injections were performed using a needle (1 inch (2.54 cm) long 30g) under electromyographic (EMG; Myoguide^®^ portable EMG machine, Bolton, ON, Canada) guidance. Kinematic tremor assessments were conducted at baseline and 6-weeks post-injection to capture the peak BoNT-A effect.

### 4.2. Kinematic Tremor Assessment

Upper limb tremor was objectively measured using motion sensor technology (three goniometers and a torsiometer; Biometrics Ltd., Newport, United Kingdom) as previously described [12,13,14]. Participants performed three trials of six scripted tasks: two rest tasks (arm in the lap with the palm facing upwards (“Rest-1”) or supported with palm facing inwards (“Rest-2”)), two postural tasks (arms outstretched with palms facing downwards (“Posture-1”) or inwards (“Posture-2”)), and two weight-bearing tasks (participants held an empty cup (“Load-1”) or a cup with a 1-pound weight (“Load-2”) in front of their chest). During the weight-bearing tasks, participants held the cup either close to their face or in front of their chest with their elbow flexed to ensure maximal tremor amplitude was captured. A variety of tasks were conducted due to changes in tremor biomechanics affected by arm position [24,25]. The kinematic tremor assessment does not extract tremor amplitudes during action movements as tremor and physiological/voluntary movements were not separated and thus action tremor was not kinematically assessed.

### 4.3. Kinematic Tremor Analysis

Kinematic datasets were analyzed using a software algorithm written in MatLab^®^ (V. 2014b, MathWorks, Natick, MA, USA) that provided tremor characteristics: amplitude of tremor represented as angular root mean squared (RMS) degrees along the whole-arm, and directional separation of tremor into planes of motion for wrist (flexion-extension (F/E), radial-ulnar (R/U), rotation/pronation-supination (P/S) deviations), elbow (F/E), and shoulder (F/E and abduction-adduction (Abd-Add)) joints.

### 4.4. Statistical Analysis

The mean change in tremor RMS amplitude (ΔRMS_follow-up-baseline_ = RMS_follow-up_ − RMS_baseline_) was plotted against the total BoNT-A dose at each arm joint for all participants. Correlations between baseline mean tremor amplitude and the change in tremor amplitude against the BoNT-A dose allocated to each joint were analyzed with Spearman-rho test (rank correlation, two-sided, *p*-value < 0.05) using SPSS^®^ statistical software (version 20, IBM^®^, Endicott, NY, USA). To investigate the relationship between the change in tremor amplitude and allocated BoNT-A joint dose per arm joint, a linear regression analysis (*p* < 0.05) was conducted.

## Figures and Tables

**Table 1:** Study demographics of selected participants treated by optimized BoNT-A injection patterns determined using clinical and kinematic guidance.

Participant ID	Condition	Arm Joint	Task to Produce Highest Tremor Amplitude	BoNT-A Joint Dose * (U)	Baseline Tremor Amplitude (RMS Degrees)	Change in Tremor Amplitude ** (RMS Degrees)
1	PD	Wrist	Rest-1	70	1.87	−0.30
2	ET	Wrist	Load-2	50	0.36	−0.29
		Elbow	Load-2	25	0.13	−0.01
3	PD	Elbow	Load-2	30	0.09	−0.01
4	ET	Wrist	Load-1	60	1.32	−1.27
5	ET	Wrist	Posture-2	70	2.27	−2.16
		Shoulder	Load-2	40	0.34	−0.32
6	PD	Wrist	Posture-1	40	0.32	−0.29
7	PD	Wrist	Rest-2	80	2.56	−1.59
8	ET	Shoulder	Load-2	200	1.07	−0.48
9	PD	Wrist	Load-2	80	2.13	−2.08
10	PD	Elbow	Load-2	40	0.32	−0.22
11	PD	Elbow	Load-2	60	0.90	−0.57
		Shoulder	Load-2	80	0.48	−0.27
12	PD	Elbow	Load-2	50	0.54	−0.40
13	ET	Shoulder	Load-2	35	0.11	−0.01
14	ET	Shoulder	Load-2	60	0.28	−0.17
15	ET	Elbow	Load-2	80	1.73	−1.45
		Shoulder	Load-2	60	0.42	−0.32
Wrist	ET: 3; PD: 4	*n* = 7	Mean ± SD	64 ± 15	1.54 ± 0.91	−1.14 ± 0.84
Elbow	ET: 2; PD: 4	*n* = 6		47 ± 20	0.62 ± 0.62	−0.44 ± 0.54
Shoulder	ET: 5; PD: 1	*n* = 6		79 ± 61	0.45 ±0.37	−0.26 ± 0.16

* Dosing optimized at the fourth serial treatment cycle; ** Change in tremor from baseline and the 6-week follow-up after the fourth injection. Abbreviations: ET: Essential Tremor; *n*: sample size; PD: Parkinson’s disease; SD: standard deviation; U: incobotulinumtoxinA (BoNT-A) units.

**Table 2:** Clinical interpretation of muscle involvement relating to the direction of tremor at each arm joint utilized in BoNT-A injection parameter determination.

Arm Joint	Dosing Equation	Degree(s) of Freedom	Muscle
Wrist	$\frac{\mathrm{Wrist}\;\mathrm{dose}\;x\;\left({\%}_{F/E\;}+{\%}_{R/U\;}\right)}{4}$	F + R	Flexor Carpi Radialis (FCR)
	$\frac{\mathrm{Wrist}\;\mathrm{dose}\;x\;\left({\%}_{F/E\;}+{\%}_{R/U\;}\right)}{4}$	F + U	Flexor Carpi Ulnaris (FCU)
	$\frac{\mathrm{Wrist}\;\mathrm{dose}\;x\;\left({\%}_{F/E\;}+{\%}_{R/U\;}\right)}{4}$	E + R	Extensor Carpi Radialis (ECR)
	$\frac{\mathrm{Wrist}\;\mathrm{dose}\;x\;\left({\%}_{F/E\;}+{\%}_{R/U\;}\right)}{4}$	E + U	Extensor Capri Ulnaris (ECU)
	$\frac{\mathrm{Wrist}\;\mathrm{dose}\;x\;\left({\%}_{P/S}\right)}{4}$	P	Pronator Teres (PT)
	$\frac{\mathrm{Wrist}\;\mathrm{dose}\;x\;\left({\%}_{P/S}\right)}{4}$	P	Pronator Quadratus (PQ)
	$\frac{\mathrm{Wrist}\;\mathrm{dose}\;x\;\left({\%}_{P/S}\right)}{2}$	S	Supinator
Elbow	$\frac{\mathrm{Elbow}\;\mathrm{dose}}{2}$	F	Biceps
	$\frac{\mathrm{Elbow}\;\mathrm{dose}}{2}$	E	Triceps
Shoulder	$\frac{\mathrm{Shoulder}\;\mathrm{dose}\;x\;\left({\%}_{F/E\;}+{\%}_{\mathrm{Abd}/\mathrm{Add}\;}\right)}{2}$	F + Add	Pectoris Major
	$\frac{\;\mathrm{Shoulder}\;\mathrm{dose}\;x{\;\%}_{F/E}}{2}$	E	Teres Major
	$\frac{\mathrm{Shoulder}\;\mathrm{dose}\;x{\;\%}_{\mathrm{Abd}/\mathrm{Add}}}{4}$	Abd	Deltoid
	$\frac{\mathrm{Shoulder}\;\mathrm{dose}\;x{\;\%}_{\mathrm{Abd}/\mathrm{Add}}}{4}$	Abd	Supraspinatus

Rotation of the forearm and wrist were grouped into the wrist/forearm dose calculation. Abbreviations: Abd = abduction, Add =Adduction, E = extension, F = flexion, P = pronation, R = radial, S = supination, U = ulnar.

## Data Availability

Data is contained within the article.
